# Is the impact of atopic disease on children and adolescents’ health related quality of life modified by mental health? Results from a population-based cross-sectional study

**DOI:** 10.1186/1477-7525-11-115

**Published:** 2013-07-08

**Authors:** Uwe Matterne, Christian Apfelbacher

**Affiliations:** 1 Department of Clinical Social Medicine, Occupational and Environmental Dermatology, University Hospital Heidelberg, University of Heidelberg, Thibautstrasse 3, 69115, Heidelberg, Germany; 2 Medical Sociology, Institute of Epidemiology and Preventive Medicine, University of Regensburg, Heidelberg, Germany

## Abstract

**Background:**

Eczema, asthma and hay fever are global health problems and their prevalence has increased considerably over the last decades. All appear to share an underlying atopic diathesis but their aetiology is considered to be multifactorial. They have been linked to decreases in health related quality of life (HRQoL) in adults, children/adolescents and/or parents of children. Research also suggests an association of the three conditions with mental health, which in turn is related to HRQoL decreases. We aimed to assess whether the impact of any of the three conditions on HRQoL is modified by presence of mental health problems.

**Methods:**

The impact of occurrence of the three conditions within the past four weeks and 12 months on HRQoL, as measured by the ‘Quality of Life in Children – Revised’ (KINDL-R) questionnaire was analysed by use of the complex sample general linear model in a population-based sample (N = 6518) of children and adolescents aged 11 – 17. Analyses were adjusted for the other atopic conditions, sociodemographic and clinical variables and stratified for mental health as measured by the Strengths and Difficulties Questionnaire (SDQ) (normal n = 5697, borderline n = 609, abnormal n = 193).

**Results:**

Eczema and hay fever within the past four weeks were significantly associated with decreased total or certain subscales of KINDL-R after adjusting for all other variables when no mental health abnormalities were present while asthma was associated with better HRQoL in these individuals. However, when mental health problems were present, eczema was positively associated with several subscales and the positive impact of asthma was stronger. The presence of mental health problems accentuated the negative relationship between hay fever and HRQoL (stronger negative impact). However, due to decreasing numbers in the group with mental health problems only few associations reached statistical significance.

**Conclusions:**

While the results suggest mental health to have a modifying effect on the relationship between some atopic conditions and HRQoL caution needs to be exercised in interpreting the results: First, the groups with borderline or abnormal mental health were comparably smaller than the group with normal mental health. In the group with normal mental health small effects were more likely to become significant than in the other two groups. Secondly some problems regarding the validity of the self-report SDQ still remain.

## Introduction

Atopic disease comprises the triad asthma, hay fever and eczema (atopic dermatitis). All are common conditions in paediatric practice [1]. *Eczema* is a very common inflammatory skin disease in children [1]. Its prevalence has been found to have increased substantially in many parts of the Western world, Asia and Africa [2] and its 12-month prevalence to be as high as 24% in some parts of the world [3]. *Asthma* is a chronic inflammatory condition of the lower airways characterised by recurrent wheezing, breathlessness and coughing [4]*Hay fever* is a chronic inflammatory condition of the upper airways that can be described by rhinorrea, nasal itching and obstruction and sneezing [5]. The lifetime prevalence in children aged 7 to 12 years may be as high as 28.4% (asthma) and 26.5% (hay fever) [6] but there is also substantial global variation [7,8].

They appear to share a common aetiological underpinning, namely that any of the three conditions is assumed to arise as a result of the interplay between the inherited atopy (predisposition) and environmental factors [9]. There is also some evidence for the so-called ‘atopic march’. The term denotes the progression from eczema to asthma and hay fever during adolescents’ development [10].

While many advances have been made in terms of identifying the multiple risk factors for the development of any of the atopic diseases, their progression and course, as well as curative and preventive interventions they still comprise very common conditions in paediatric practice [1] and have been consistently linked to decreases in health-related quality of life (HRQoL) [11-14]. Consequently, pediatric HRQOL has emerged as an important health outcome in clinical trials and healthcare research [15]. Several studies have also observed an association of mental health problems with HRQoL [16-20]. A previous analysis [21] was able to show that several domains of HRQoL were significantly impaired even after control for mental health problems in children and adolescents with eczema, asthma or hay fever. These analyses also took atopic comorbidity into account, i.e. controlled for the effects of other atopic disease on HRQoL. This allowed capturing the impact of each single condition independent of any existing atopic comorbidity and any mental health problems.

Due to the chronicity of atopic disease adequate adaptation appears necessary in several domains. Because the ability to successfully adapt may vary as a function of mental health it is plausible to expect a higher impact of any disease but also atopic disease on HRQoL in individuals with mental health problems. This higher impact may be seen as the result of fewer perceived resources, more perceived stress or less self-efficacy to name but a few dimensions. Modifying effects of mental health on the relationship between disease and HRQoL were demonstrated for various chronic conditions [22-25]. Some studies even showed that most of the impact of a chronic condition on HRQoL was confined to cases with co-morbid mental disorders [26]. However, no data is available with respect to whether the level of HRQoL impairment varies as a function of mental health problems in children and adolescents suffering from atopic disease.

It was the aim of this analysis to examine whether the impact of eczema, asthma and hay fever occurrence (both within the past four weeks and the past 12 months) varies as a function of mental health status. It was expected that the presence of mental health problems would lead to more pronounced impairments in HRQoL across atopic disease but that acuteness (4-week versus 12-month occurrence) would also be associated with differential effects. A second aim was to identify the HRQoL-areas which are particularly affected in groups formed on the basis of mental health status.

## Method

### Design and participants

We analysed the public use files of the KiGGS (Health Interview and Examination Survey for Children and Adolescents) study [27]. The study was approved by the Federal Office for Data Protection and by the ethics committee of the university hospital Charité, Berlin [28]. KiGGS, which is a representative cross-sectional survey, was conducted from 2003 to 2006 in 167 sampling units (locations) in Germany. Within each sampling unit participants were randomly selected from the local population registries. From a total of 28,299 individuals invited 17,642 aged 0–17 years agreed to participate (response rate: 66.6%). A detailed description of the two-stage sampling method is provided elsewhere [29]. Of the 17,642 individuals taking part in the KiGGS, 6,813 were children and adolescents aged 11 – 17 years. The present analyses are based on 6,518 individuals for whom data on KINDL-R (Quality of Life in Children - Revised [30,31] and the atopic conditions was available. 2935 (45%) were aged 11 – 13 years and 3583 (45%) aged 14 – 17 years.

### Instruments

Data from the KiGGS used in this investigation are based on self-administered questionnaires completed by parents/caregivers, computer assisted personal interviews (CAPI) with parents/caregivers and children/adolescents’ self-reports of HRQoL. The CAPI was conducted by physicians who were specifically trained for the purposes of the study and, besides vaccination status and medication use within the past 7 days, assessed the history of selected physician-diagnosed conditions. If parents gave their consent, a blood sample was taken. Using the system IMMUNOCAP (Phadia), specific IgE was determined in those children aged 3–17 years for the following antigenes: Dermatophagoides pteronyssinus, Dermatophagoides farinae, cat dander, horse epithelium, dog dander, egg white, peanut, soy bean, milk protein, carrot, potato, wheat flour, green apple, rice, rye pollen, timothy grass pollen, Cladosporium herbarum, Aspergillus fumigatus, birch pollen, mugwort pollen [32].

The 4-week and the 12-month prevalence of eczema, asthma and hay fever/allergic conjunctivitis were based on parents’ reports of a medical diagnosis made by a physician within the past four weeks or 12 months, respectively in the CAPI.

HRQoL was measured by the German KINDL-R questionnaire which contains 24 items [30]. Its psychometric properties have been established [31]. Generic in nature it measures the following six dimensions of quality of life: Physical well-being (PWB), emotional well-being (EWB), self-esteem (SE), family (quality of relationship with family), friends (quality of relationship with friends), and everyday-functioning (well-being at school). The recall period is the past 7 days. It accounts for differences in development by providing different versions for different age groups. Items are scored on a 5-point Likert scales (never, seldom, sometimes, often, always). For each dimensions a total score can be computed by adding the respective items forming that dimension. An overall total KINDL-R score is computed by summing all 24 items. All scores are transformed into values ranging from 0 – 100. Larger scores denote better quality of life. For the present analysis the self-report version of the KINDL-R was used.

The database used for the present analyses further contains information (assessed by parent report) on age in years, gender (male vs. female), migration status (German versus migration background), weight and height. The latter two were used to compute the Body-Mass-Index which was used to classify overweight according to Krohmeyer-Hausschild [33]. It further contains data (education, occupation and family income) which is used to create the composite socioeconomic status (SES; low, medium, high) [34]. Finally, allergic sensitisation was operationalised as yes (sensitization to at least one of the above food or aero-allergens) or no. All analyses were adjusted for these variables. Covariates were chosen based on evidence regarding their relationship with HRQoL and/or the atopic conditions.

The self-report version of the Strengths and Difficulties Questionnaire (SDQ) [35], a brief 25-item behavioural screening questionnaire assessing emotional symptoms, conduct problems, hyperactivity/inattention, peer relationship problems and prosocial behaviour was used to provide a measure of mental health (normal, borderline, abnormal). Individual SDQ-items are scored on a 3-point scale (0 not true, 1 somewhat true, and 2 certainly true); higher scores denote greater problems except for prosocial behaviour, for which a higher score is suggestive of more positive behaviour. All items apart from the prosocial behaviour items are used to calculate a total score (range 0 – 40) with higher scores indicating more problematic behaviour. According to the cut-offs provided [36] individuals are assigned to one of the three groups (≤ 15 = normal, > 15 = borderline, > 19 = abnormal mental health). Although the self-report SDQ’s reliability and validity have been established across cultures to some extent [37], it has to be noted that the SDQ fares less well than a full clinical interview in terms of validity [38].

### Statistical analysis

Data was analysed using SPSS 20 (IBM Statistics). A cluster variable was specified to account for non-independence of observations within the 167 clusters (sampling points) from which individuals were recruited. This was done to give us greater confidence in accepting a significant effect as being a population parameter. Failing to model the design effect (clustering) would lead to an underestimation of the respective standard errors and thus lead to less valid conclusions about the data. Data was also weighted according to a weight factor correcting for deviations within the sample from the population structure in terms of age, gender, region and nationality [29]. By doing so, the findings become applicable to the population of German children and adolescents at large. SPSS’ GLM (General Linear Model) procedure for complex samples was used to analyse the associations of the three conditions with HRQoL in multivariate analysis within each category of mental health status while controlling for other atopic comorbidity, age, gender, ethnic origin, socioeconomic status, overweight and allergic sensitization. This procedure yields unstandardised *Beta* coefficients (*B*). These can be interpreted as increments in the dependent variable (in the original units of measurement) in individuals in whom the condition is present in comparison to those individuals in whom the condition is not present holding all other variables constant.

## Results

Sociodemographic and clinical characteristics are displayed in Table 1. The epidemiological parameters of the sample as well as the main effects of the three atopic conditions on HRQoL are described in detail by Matterne et al. [21]. In brief, the vast majority did not show signs of mental health problems (SDQ = 0: 87.7%; SDQ = 1: 9.5%; SDQ = 2: 2.8%) and the four-week prevalence of eczema was 4.3%, of asthma 2.4% and of hay fever 5.1%.

**Table 1:** Sample description: absolute and relative frequencies of sociodemographic and clinical characteristics (N = 6518)

	***n***	***%***	***%***^***a***^	**95*% CI ***^***a***^	
**Age** (*n* = 6518)					
11 – 13 years	2935	45.0	39.7	38.8	40.5
14 – 17 years	3583	55.0	60.3	59.5	61.2
**Gender** (*n* = 6518)					
Male	3326	51.0	51.1	50.7	51.4
Female	3192	49.0	48.9	48.6	49.3
**Origin** (*n* = 6517)					
German	5530	84.9	83.0	80.7	85.1
Migration Background	987	15.1	17.0	14.9	19.3
**SES** (*n* = 6309)					
Low	1697	26.9	27.2	25.7	28.7
Medium	3057	48.5	47.3	45.8	48.7
High	1555	24.6	25.6	23.9	27.3
**Overweight** (*n* = 6491)					
Yes	1152	17.7	17.6	16.6	18.7
No	5339	82.3	82.4	81.3	83.4
**Allergic Sensitisation** (*n* = 6107)					
Yes	2763	45.2	45.8	44.2	47.3
No	3344	54.8	54.2	52.7	55.8

*SES* Socioeconomic status, ^a^weighted percentages simultaneously accounting for nested structure (sampling points).

Table 2 displays the results of the multivariate analyses regressing eczema, asthma and hay fever (once within the past 4 weeks and once within the past 12 months, respectively) on total KINDL-R and its subscales’ scores stratified by mental health and controlled for presence of other atopic condition as well as age, gender, origin, SES, overweight and allergic sensitisation.

**Table 2:** Analyses of impact of atopic condition (within past 4 weeks and 12 months) on total and subscales of KINDL-R stratified by mental health and controlled for presence of other atopic condition as well as age, gender, origin, ses, overweight and allergic sensitisation

	**Within past 4 weeks**			**Within past 12 months**		
	**SDQ = 0**	**SDQ = 1**	**SDQ = 2**	**SDQ = 0**	**SDQ = 1**	**SDQ = 2**
	**(n = 5149)**	**(n = 536)**	**(n = 158)**	**(n = 5120)**	**(n = 536)**	**(n = 158)**
KINDL-R Total						
Eczema	−1.82^*^	−0.98	2.80	−1.07	−0.03	2.28
Asthma	0.92	−1.49	3.04	0.08	−0.60	0.70
Hay fever	−1.46^*^	−3.98	−10.66	−1.14^*^	−1.84	−1.63
KINDL-R Physical						
Eczema	−2.21^*^	−5.43	10.47	−1.97	−5.19	7.49
Asthma	−0.13	−7.56	4.56	−1.80	−4.79	1.29
Hay fever	−4.96^***^	−3.30	−11.31	−2.44^**^	−4.34	−2.23
KINDL-R Emotional well-being						
Eczema	−1.53	−3.89	1.69	−0.28	−0.69	2.87
Asthma	−0.14	−2.34	6.82	−0.20	−2.07	5.57
Hay fever	−0.31	−5.38	−19.07^*^	−0.29	−0.63	−4.82
KINDL-R Self						
Eczema	−3.01^*^	3.93	−4.08	−1.10	6.39	−5.37
Asthma	1.39	−6.17	−1.37	0.49	−1.78	−6.12
Hay fever	−0.67	0.05	−6.89	−1.21	−1.37	3.49
KINDL-R Family						
Eczema	−1.37	−2.13	10.44^*^	−2.36^*^	−1.09	6.52
Asthma	0.26	0.73	3.75	0.40	−1.09	0.29
Hay fever	−1.65	−4.75	−8.12	−1.17	−1.17	−2.67
KINDL-R Friends						
Eczema	−0.60	1.69	3.26	0.41	2.08	6.65
Asthma	0.19	1.50	5.87	0.11	−0.81	4.55
Hay fever	−1.30	−8.56	−4.88	−0.70	−5.30	−1.80
KINDL-R School						
Eczema	−2.17	−0.35	−4.91	−1.04	−2.00	−4.77
Asthma	3.85^*^	5.03	−0.44	1.27	6.69	−0.20
Hay fever	−0.60	−0.52	−11.21	−1.22	2.56	−0.89

* p ≤ 0.05, ** p ≤ 0.01, *** p ≤ 0.001, SDQ = 0: normal, SDQ = 1: borderline, SDQ = 2: abnormal mental health, numbers in table refer to unstandardised *Beta* coefficients (*B*) that can be interpreted as increments in KINDL-R scores in individuals in whom the condition is present compared to those in whom the condition is not present while holding all other variables constant.

Eczema within the past four weeks was significantly associated with decreases in total KINDL-R scores when no mental health abnormalities were present. Presence of eczema in this group led to a 1.82 point decrease in total HRQoL holding all other variables constant. Most of this decrease was due to impairments in the physical and self-worth (self-esteem) domain. With increasing levels of mental health abnormalities no significant associations of eczema within the past four weeks and KINDL-R scores were observed apart from a significant association of eczema with the family domain in the group with mental health abnormalities. Here presence of eczema led to a 10.44 point improvement in the quality of the relationship with one’s family while controlling for all other variables. Further, the relationship’s direction was reversed in the group with abnormal mental health for the total and four of the six subscales.

Asthma within the past four weeks was significantly related to better well-being at school but no other significant relationship was observed in the group with no mental health abnormalities. No significant associations with asthma within the past four weeks were observed in the group with borderline or in the group with mental health abnormalities.

In the group with no mental health abnormalities hay fever within the past four weeks was significantly related to reduced total KINDL-R scores and the impact was mainly due to impairments in physical functioning. In the group with mental health abnormalities, total and subscale scores were consistently and markedly lower compared to the group with no mental health abnormalities. However, a significant effect of hay fever on KINDL-R scores was only found for emotional well-being in this group. Hay fever within the past four weeks reduced children’s emotional well-being by almost 20 points while controlling for all other variables.

With regard to the impact of the three atopic conditions within the past 12 months on HRQoL it was obvious that the impact was less pronounced compared to the 4-week occurrence and that consequently fewer associations reached significance. The only significant associations were observed for eczema on the family relations subscale and of hay fever on total and the physical functioning subscale in the group with no mental health abnormalities. The direction of association was negative. No significant associations were detected in the other two groups. However, similar to the 4-week occurrence of eczema in the group with mental health abnormalities the associations, albeit insignificant, were reversed for total and four subscales’ scores of KINDL-R. More impairment in total and three subscale scores was observed for the group with mental health abnormalities in comparison with the group with no mental health problems but the differences were markedly lower compared to the 4-week occurrence of hay fever.

## Discussion

We aimed to assess whether the impact of any of the three atopic conditions is modified by concurrent levels of mental health problems. The rationale behind this was to obtain information that can be taken into account when evaluating the impact of any of the three conditions on HRQoL in an individual. This may then lead to different judgements as to what areas need to additionally or particularly be targeted to help certain children or adolescents to better come to terms with their atopic condition(s).

The present analyses clearly revealed the associations of any of the three atopic conditions on children and adolescents’ HRQoL to vary as a function of mental health status. However, only few of the relatively large effects in the group with abnormal mental health reached significance. This is likely the result of the decreasing sample size corresponding with increasing levels of mental health problems. The sample size decreased from 5697 (no abnormalities), to 609 (borderline) to 193 (abnormal mental health). In other words, with respect to the group of individuals with mental health problems the present analyses were afflicted with a statistical power problem. This poses a major dilemma because while KiGGS is a large population based survey it appears as if for certain analyses even that size may not have been sufficient to give the power necessary for certain statistical tests. The number of valid inference-based conclusions to be drawn is thus limited. However, part of the results can be viewed in an exploratory fashion offering ample scope for future research. For instance, because of the large sample that is necessary to obtain a sufficient number of individuals with mental health abnormalities it might be an option to recruit a sufficiently large number of individuals with mental health abnormalities and compare their HRQoL with a control group with no mental health problems that is matched for all other characteristics with the group with mental health problems. Further, it has to be pointed out, that diagnostic information was based on parent report of physician diagnosed atopic disease, which poses the typical problems self-report can be afflicted with (see limitations below).

A main finding of the present analyses is that any of the three atopic conditions impacts on HRQoL in children and adolescents when no mental health problems are present. The specific impact of each condition varies, however [see [21].

In the group with mental health abnormalities, eczema paradoxically appears to have a positive effect on some domains of HRQoL. Those with eczema within the past four weeks had a significant 10 point increase in the family relation domain. It may be that the presence of eczema binds the family more closely together thus overcoming the often destructive character of mental health abnormality on family relations. Alternatively, parents and siblings may be more accepting of a child’s behaviour if it can be attributed to a physical rather than a mental health problem which may in turn relax family relations. Mental illness is still associated with substantial stigma [39] and there is still a widespread assumption that physical illness is more legitimate than mental illness [40] and as thus relatives of sufferers may be more accepting and more supportive. In other words, children and adolescents with eczema may encounter more support during flares from their families and thus view the impact on the family not as impairing but as facilitating. More research, however, is necessary to disentangle the exact dynamics behind this observation. A similar but less pronounced trend was observed with regard to asthma. The combination of acute asthma (past four weeks) and mental health abnormalities was associated with better total and some subscale scores of KINDL-R. However, the group size was again too small for these effects to reach statistical significance. While children and adolescents with asthma have been found to be at increased risk for mental health problems, particularly internalising problems (emotional well-being) [41] leading to suggestions of accumulative risks for HRQoL-impairments [42] it may also be necessary to look at the amount of external resources available (e.g. social support). It may be that these resources are provided more when asthma and mental health problems coincide and then HRQoL is not affected in cumulative fashion but support can help individuals better come to terms with both conditions but again, more research is needed.

Acute hay fever (past four weeks), however, was associated with stronger HRQoL-impairment in individuals with mental health problems in comparison to those with no such problems. For this disease it appears as if the burden is increased when it is paired with mental health problems. Nevertheless, perhaps due to limited power, only the effect on emotional well-being reached statistical significance but the decrements in HRQoL were quite substantial. When hay fever attacks occur in an individual already affected by mental health problems these additional problems may have an accumulative character unlike what was observed with regard to eczema or asthma. Hay fever is a seasonally occurring condition in contrast to eczema which although characterised by flares can appear all year round. The modifying effect of mental health was mainly limited to acute hay fever (past four weeks). No substantial or significant effects were observed for hay fever within the past 12 months. As hay fever is highly seasonal this finding indicates that most of the effect of hay fever on HRQoL is immediate rather than persistent over time, irrespective of the presence of additional mental health problems.

In addition to the discussed power issue, the findings also need to be viewed in light of how valid atopic disease and mental health were assessed in this study. Both were assessed by self-report which is often afflicted with an information bias due to the well-known problems of social desirability or memory distortion. Moreover, there is also the problem of how constructs are operationalised. For instance, while the SDQ is widely used it has to be borne in mind that it is after all a screening instrument for mental health status and secondly that concerns have been raised with regard to misclassification resulting from inadequate validity [37,38].

## Conclusion

While it is generally believed that mental health problems accentuate the relationship between chronic conditions and HRQoL the present analyses found a diverse pattern of relationships between any of three atopic conditions on HRQoL when analyses were conducted separately as a function of concurrent mental health problems. Future studies should not only consider any joint effects of several diseases and concurrent mental health problems, but also what extra-personal resources are available and how they may influence the impact of the atopic condition on HRQoL in order to draw a clearer picture of how combinations of conditions influence HRQoL differently.

## Competing interests

The authors declare that they have no competing interests.

## Authors’ contributions

UM and CA devised the conception and design of the study. Statistical analyses were carried out by UM. The interpretation of the findings occurred co-jointly between UM and CA. The manuscript was drafted by UM. UM and CA critically revised the manuscript for important intellectual content. Both authors gave their final approval of the version to be published/submitted.
